# Causal Linkage Between Inflammatory Bowel Disease and Primary Sclerosing Cholangitis: A Two-Sample Mendelian Randomization Analysis

**DOI:** 10.3389/fgene.2021.649376

**Published:** 2021-03-18

**Authors:** Ying Xie, Xuejie Chen, Minzi Deng, Yuhao Sun, Xiaoyan Wang, Jie Chen, Changzheng Yuan, Therese Hesketh

**Affiliations:** ^1^Centre for Global Health, Zhejiang University School of Medicine, Hangzhou, China; ^2^Department of Gastroenterology, The Third Xiangya Hospital, Central South University, Changsha, China; ^3^Department of Big Data and Health Science, Zhejiang University School of Medicine, Hangzhou, China; ^4^Institute for Global Health, University College London, London, United Kingdom

**Keywords:** inflammatory bowel disease, ulcerative colitis, Crohn’s disease, mendelian randomization, primary sclerosing cholangitis

## Abstract

**Background:**

Observational studies suggest an association between inflammatory bowel disease (IBD) [including ulcerative colitis (UC) and Crohn’s disease (CD)] and Primary sclerosing cholangitis (PSC), but the causal association between the two diseases remains unclear.

**Methods:**

We used two-sample Mendelian randomization (MR) to estimate the causal association between IBD and PSC. We chose single nucleotide polymorphisms (SNPs) data for analysis, obtained from previous genome-wide association studies (GWASs). Pleiotropy, heterogeneity, and sensitivity analyses were performed for quality control.

**Results:**

We found that the causal associations between IBD (both UC and CD) and PSC were significant (e.g., IBD and PSC, Robust adjusted profile score (RAPS) OR = 1.29, 95% CI 1.16∼1.44, *p*< 0.01; UC and PSC, RAPS OR = 1.40, 95% CI 1.23∼1.58, *p*< 0.01; CD and PSC, RAPS OR = 1.13, 95% CI 1.02∼1.26, *p* = 0.02). MR Egger, IVW, and ML tests found statistical heterogeneity between determined IV estimates. The leave-one-out analysis also indicated the sensitivity of the SNPs (e.g., IBD and PSC, MR-Egger Q = 644.30, *p*< 0.01; UC and PSC, MR-Egger Q = 378.30, *p*< 0.01; UC and PSC, MR-Egger Q = 538.50, *p* < 0.01).

**Conclusion:**

MR analyses support the positive causal effect of IBD (including UC and CD) on PSC in a European population. We provide suggestions for preventing and treating the two diseases.

## Introduction

Primary sclerosing cholangitis (PSC) is a rare, progressive cholestatic disease featuring impaired bile formation and chronic liver dysfunction, led by inflammation and fibrosis with a 0.77 per 100,000 person-years incidence rate (Molodecky et al., 2011; Karlsen et al., 2017; Dyson et al., 2018). Both genetic and environmental factors contribute to PSC, with the intestinal microbiome being considered as a pathogenetic factor. Inflammatory bowel diseases (IBDs) describe a series of chronic inflammatory disorders of the gastrointestinal tract including two main types: Crohn’s disease (CD) and ulcerative colitis (UC) (Rosen et al., 2015; Hodson, 2016).

It has been reported that IBD and PSC are closely associated. According to a comprehensive review, the prevalence of IBD in PSC has reached two-thirds (Karlsen et al., 2017). It has been observed that total colectomy can reduce the recrudesce risk of PSC by 50%, prior to or within liver transplantation (Lindström et al., 2018; Ricciuto et al., 2018). It has also been reported that the inflammatory type of PSC differs from UC or CD (Karlsen et al., 2017). Genetically, PSC appears to be more like an autoimmune condition compared with IBD (Liu et al., 2013). Although the striking association has been found for more than 50 years (Warren et al., 1966), the mechanisms for the relationships between the two diseases remain elusive.

The causal relationship between IBD and PSC is important in exploring the function of the disease, and thus in informing evidence for effective treatment. Randomized controlled trials (RCTs) are the most reliable method for determining causal inference in treatment studies. However, due to the requirements of the design and implementation, difficultly to control, and the consideration of ethics, RCTs are difficult to conduct. We used the Mendelian randomization (MR) analysis to explore the likely causal relevance between exposure and outcome, based on observational epidemiological studies.

Because gametes follow Mendelian rules of inheritance (parental alleles are randomly assigned to offspring), genetic variation is not affected by confounders such as environmental exposure, socioeconomic status, and behavior. Furthermore, genetic variation comes from parents, thus the association with outcomes is chronological. Therefore, MR can overcome the problems of confounding and reverse causality.

The instrumental variables (IVs) in the MR study rely on three core assumptions: (a) the genetic variant (either combined or isolated with other variants) is associated with the exposure; (b) the genetic variant is not associated with confounders that are either known or unknown; (c) there is no pathway from the genetic variant to outcomes that do not include the exposure. In MR research, it is difficult to obtain an accurate estimate of causal association without any one of the above assumptions.

Genome-wide association studies (GWAS) featuring large sample sizes make genetic variants available. Based on the previous GWAS, we selected single nucleotide polymorphisms (SNPs) that are strongly relevant to IBD (including UC and CD) as IVs. The effect of IVs on the exposures (IBD) and outcomes (PSC) was from two independent samples. We used two-sample MR and statistical methods to analyze the quantitative effects of IBD (UC, CD) on PSC.

## Materials and Methods

### Data Source

More SNP sites related to IBD were screened out by GWAS results combined with literature reports. This study chose SNPs from publicly available GWAS data bases associated with exposures (IBD, including UC and CD). IBD-associated SNPs were derived from a GWAS meta-analysis study of IBD in the European Genome-phenome Archive (EGA). The statistics came from an extended cohort of 86,640 European individuals and 9,846 non-Europeans (Liu et al., 2015). Studies showed that most of the risk loci were shared across divergent populations (Teslovich et al., 2010; Okada et al., 2014; Liu et al., 2015). The SNPs associated with PSC were selected from the largest GWAS of PSC up to date, the European population, including 4,796 cases and 19,955 population controls. Quality control, like the Inverse variance weighted (IVW) fixed-effects meta-analysis, was performed to test the evidence of association across the GWAS and cohorts. Bayesian tests were conducted in both studies to identify loci with strong evidence.

### SNP Selection

From the collection of SNPs in previous studies, we then set some standards for including eligible SNPs. We chose SNPs that were significantly associated with exposures (*p* ≤ 5e-8) and that had a certain probability of mutation (Minor allele frequency, MAF ≥ 5%), without reported loci coincidence or linkage disequilibrium (LD) (*R*^2^<0.001). The palindromic SNPs which can introduce ambiguity into the identity of the effect allele in the exposure GWAS were also excluded. The SNPs that were both related to PSC and IBD were the excluded to meet the third core assumption, eliminating other pathways from the genetic variant to outcomes that do not include the exposure.

### Effect Size Estimate

**FIGURE 1:**
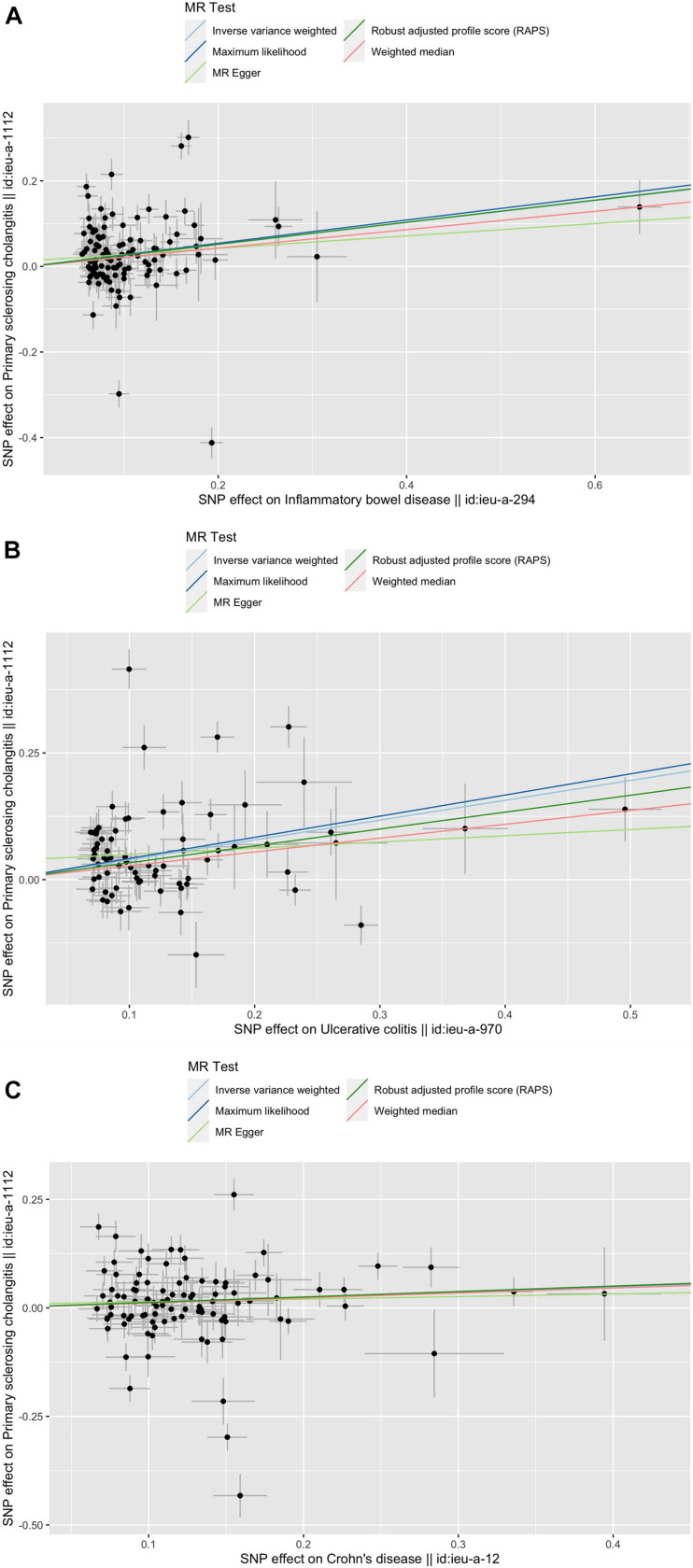
Scatter plots of the genetic causal associations with IBD, UC, and CD against PSC using different MR methods. **(A)** IBD against PSC; **(B)** UC against PSC; and **(C)** CD against PSC. The slopes of the line represent the causal association for different methods. The light green line represents the MR Egger, the dark green line represents the Robust adjusted profile score (RAPS), the pink line represents the weighted median estimate, the light blue line represents the Inverse variance weighted estimate, and the dark blue line represents the Maximum likelihood estimate.

We estimated the causal association between exposures (IBD, UC and CD) and outcomes (PSC) with Inverse variance weighted (IVW), MR Egger, Weighted median (WM), Robust adjusted profile score (MR. RAPS), and Maximum likelihood (ML). IVW takes the inverse variance of each study as the weight to calculate the weighted average of effect sizes, to summarize the effect sizes of multiple independent studies (Lee et al., 2016). We also performed the Weighted median estimator (WME), with which causal effects can be accurately estimated with more than 50% weight using IVs when doing the analysis (Bowden et al., 2016a). A newly developed analysis called Robust adjusted profile score (MR. RAPS) considering the measurement error in SNP-exposure effects was conducted to reduce bias from weak IVs (Zhao et al., 2019). Maximum likelihood maximizing the likelihood function to estimate the probability distribution parameters was also used as a reference traditional method (Milligan, 2003). However, due to potential pleiotropic effects, the genetic variants may influence the outcome in an additional way, thus causing a bias. Therefore, we used MR-Egger regression as well, the slope of which can estimate the magnitude of directional pleiotropy. The MR-Steiger directionality test is used to test the causal direction between the hypothesized exposure and outcomes as a verification of the reliability of the results (Hemani et al., 2017). The results were presented in odds ratios (OR) and 95% confidence intervals (CI). A two-sided *p*-value was considered statistically significant when it was less than 0.05 (Figure 1). All statistical analyses were performed in R 3.4.2 with the package “TwoSampleMR.”

### Sensitivity Analyses

In the MR analysis, it is necessary to consider whether SNPs, as instrumental variables, associate with other exposures. We used MR-Egger to test pleiotropy, verifying whether a single locus affects multiple phenotypes. The “Leave-one-out sensitivity analysis” algorithm is used as sensitivity analyses. With non-specific SNPs eliminated, if the correlation between other instrumental variables and outcomes was still statistically significant, it indicates more sufficient evidence of the causal association between exposure and outcomes. By removing SNPs one by one, the results are reanalyzed to draw the forest map with a stable result intuitively judged (Supplementary Figures S1–S3). As for the heterogeneity analysis, we conducted it for MR Egger, Inverse variance weighted, and Maximum likelihood. Heterogeneity was standardized with Cochran Q statistics; the weighted sum of the squared differences between the effect of each SNP and the summed effect of all SNPs. We also used a two-sided *p*-value and considered statistical significance at *p* < 0.05.

## Results

Based on the selection criteria above, we conducted linkage disequilibrium tests to choose SNPs that are both related to IBD and PSC. A total of 121, 76, and 104 SNPs were selected as IVs for IBD, UC, and CD, respectively. We then excluded 17 palindromic SNPs (nine for IBD, three for UC, and five for CD). Finally, we included 112, 73 m and 99 SNPs for IBD, UC, and CD, respectively (Supplementary Tables S4–S6).

The causal associations between IBD (UC, CD) and PSC were not accordant among the five methods. The RAPS indicated that IBD (both UC and CD) was significantly associated with PSC (IBD and PSC, RAPS OR = 1.29, 95% CI 1.16∼1.44, *p*< 0.01; UC and PSC, RAPS OR = 1.40, 95% CI 1.23∼1.58, *p*< 0.01; CD and PSC RAPS OR = 1.13, 95% CI 1.02∼1.26, *p* = 0.02) (Table 1). However, using MR-Egger, none were significantly associated with PSC (For IBD, OR = 1.16, 95% CI 0.82∼1.63, *p* = 0.41; For UC, OR = 1.13, 95% CI 0.80∼1.61, *p* = 0.50; For CD, OR = 1.06, 95% CI 0.75∼1.50, *p* = 0.74) (Table 1). When all genetic variants are valid, the causal effect may be underestimated due to the inflated type I error (Bowden et al., 2016b). Additionally, ML, WM showed the significant associations between UC, CD, and PSC while IVW did not reveal the associations between CD and PSC (Table 1). Based on the above five analyses, we concluded that the causal association between IBD and PSC were significant.

**TABLE 1:** MR estimates from each method assessing the causal effects of UC, CD, and IBD on PSC.

Exposure traits	MR methods	PSC				
		Number of SNPs	OR(95% CI)	SE	MR *p*-value	MR-Steiger test
IBD	MR Egger	112	1.16 (0.82∼1.63)	0.17	0.41	TRUE
	Inverse variance weighted	112	1.29 (1.12∼1.50)	0.08	<0.01	
	Maximum likelihood	112	1.31 (1.23∼1.34)	0.03	<0.01	
	Weighted median	112	1.24 (1.10∼1.40)	0.06	<0.01	
	Robust adjusted profile score (RAPS)	112	1.29 (1.16∼1.44)	0.05	<0.01	
UC	MR Egger	73	1.13 (0.80∼1.61)	0.18	0.50	TRUE
	Inverse variance weighted	73	1.48 (1.27∼1.72)	0.08	<0.01	
	Maximum likelihood	73	1.52 (1.42∼1.63)	0.04	<0.01	
	Weighted median	73	1.31 (1.15∼1.49)	0.06	<0.01	
	Robust adjusted profile score (RAPS)	73	1.40 (1.23∼1.58)	0.06	<0.01	
CD	MR Egger	99	1.06 (0.75∼1.50)	0.18	0.74	TRUE
	Inverse variance weighted	99	1.13 (0.99∼1.28)	0.07	0.07	
	Maximum likelihood	99	1.13 (1.07∼1.20)	0.03	<0.01	
	Weighted median	99	1.12 (1.02∼1.24)	0.05	0.02	
	Robust adjusted profile score (RAPS)	99	1.13 (1.02∼1.26)	0.05	0.02	

Pleiotropy, heterogeneity, and sensitivity analyses were performed for quality control. We used MR-Egger regression to test the pleiotropy, finding an unlikely bias caused by horizontal pleiotropy (IBD *p* = 0.48, UC *p* = 1.03, CD *p* = 0.72) (Table 2). To test the heterogeneity, we conducted MR Egger,IVW, and ML, finding statistical heterogeneity between determined IV estimates(e.g., For IBD, MR-Egger Q = 644.30, *p* < 0.01; For UC, MR-Egger Q = 378.3, *p* < 0.01; For UC, MR-Egger Q = 538.50, *p* < 0.01.)(Table 2). For sensitivity, we conducted a Leave-one-out sensitivity analysis, finding that the MR estimates were reasonable considering the effect of single SNPs (Supplementary Figures S1–S3). Additionally, the MR-Steiger test supported a positive causal correlation between IBD (UC, CD) and PSC, also identifying IVs’ affecting susceptibility to IBD traits and PSC. These results indicate the powerful relevance of MR assumption and the weak bias in the analysis.

**TABLE 2:** Heterogeneity and pleiotropy analysis of UC, CD, and IBD with PSC, using different analytical methods.

Exposure traits	MR methods	PSC		
		Cochran Q statistic	Heterogeneity *p*-value	Pleiotropy *p*-value
IBD	MR Egger	644.30	<0.01	0.48
	Inverse variance weighted	647.28	<0.01	
	Maximum likelihood	643.80	<0.01	
UC	MR Egger	378.30	<0.01	0.10
	Inverse variance weighted	392.85	<0.01	
	Maximum likelihood	386.16	<0.01	
CD	MR Egger	538.50	<0.01	0.72
	Inverse variance weighted	539.21	<0.01	
	Maximum likelihood	538.40	<0.01	

## Discussion

To our knowledge, it is the first study to illustrate the causal relationship between IBD (UC, CD) and PSC using MR and GWAS. We found that IBD (including UC and CD) had a causal association with PSC, indicating that they may have a similar pathogenesis.

Several hypotheses have been proposed over the years, to explain the mechanisms of the model (Karlsen, 2016). An early RCT found that a small bowel bacterial overgrowth is associated with bile duct proliferation and destruction. Hypotheses based on the “leaky gut concept” indicated that bacteria and bacterial products could pass through damaged mucosa in IBD into the portal circulation (Lichtman et al., 1991). A review also reported that gut-derived mucosal T-cells expressing α4β7 would contribute to biliary inflammation. Barrier functions like the expression of pathogen pattern receptors are similar between the biliary and gut epithelium. The receptor CXCR6, is found to have a higher expression on liver-infiltrating and gut-infiltrating lymphocytes. Blocking the receptors is a developing treatment for inflammation (Adams et al., 2008). Another hypothesis suggests the possibility of FtsZ and TBB-5 antigens deriving from colonic content, which may drive the biliary inflammation. This is related to an abnormal immune response to intestinal microorganisms in susceptible individuals (Terjung et al., 2010). In summary, the association may result from hyperreactive bile duct proliferation, aberrant increased enterohepatic circulation pathogen-associated molecular patterns (PAMPs), or an abnormal immune response (Tabibian et al., 2013).

All five MR methods indicated a significant relationship between UC and PSC. Another study also reported the strong association of PSC with UC (90%) (Adams et al., 2008). As for CD, MR Egger, IVW showed no significant relationship between CD and PSC, while the other three methods revealed the causal relationship. MR Egger and IVW are similar, both using the inverse of the outcome variance (Se^2^) as the weight to carry out the fitting. The biggest difference between them is whether or not to consider the intercept term in the regression. Because of the low statistical power of MR-Egger, we usually focus on the consistency of the direction rather than the significance of estimates (Yeung and Schooling, 2020). From Supplementary Figures S1–S3, the consistent direction can be intuitively judged. Thus, we conclude that both UC and CD have a significant relationship with PSC.

In the Leave-one-out sensitivity analysis, we also found the specific SNPs that are strongly related to the disease (rs9836291 and rs2836883 for IBD; rs9836291 and rs2836883 for UC; rs3197999for CD). A previous study reported that the chromosome 3 SNP (rs3197999) is in the MST1 (Macrophage Stimulating 1) gene and is associated with MST1 protein levels. This SNP (rs3197999) can induce IBD by regulating the protein level of the Macrophage Stimulating Protein (MSP) (Di Narzo et al., 2017). Our results may provide inspiration for possible mechanism analyses in the future.

The causal association of IBD and PSC could contribute to improvement in PSC diagnostics and therapy, as well as prevention for IBD patients. For PSC, the diagnostics and therapy should better include IBD as a factor for improvement. According to PSC guidelines in the United States and Europe (Valuing Integrity, 2009; Lindor et al., 2015), major detection includes markers of cholestasis, bile duct lesions, and structuring on cholangiography with Magnetic resonance cholangiopancreatography (MRCP), along with a liver biopsy. Apart from these diagnostic investigations, we suggest regular colonoscopy surveillance for detecting IBD. For PSD patients with or without IBD, the clinical treatment and follow-up may be different. For example, clinical trials have tested the positive effect of antibiotics in PSC treatment (Tabibian et al., 2013). However, we should consider the potential consequent disturbance of gut microbiota (Karlsen, 2016), especially for IBD patients. For IBD, measures should be taken to prevent PSC at the very beginning. PSC-IBD has become an important public health issue due to the increased risk of malignancy (Rossi et al., 2016). Thus, regular physical examinations of PSC signs and symptoms are necessary for IBD patients. Currently, gut microbial signatures have been reported for their discriminatory function of determining early-stage PSC in IBD (Tabibian et al., 2013; Karlsen, 2016). Admittedly, the complex physiological machinery between IBD and PSC goes far beyond such simple models. Further studies are also needed to identify a potential mechanism for the association between IBD and PSC, to inform disease prevention.

Our study has several limitations. First, the SNP statistics we used were from a mixed population, 89.8% (86,640 in 96,486) of Europeans. However, the selected SNPs can explain 0.085 for IBD, 0.044 for UC, and 0.105 for CD of the phenotypic variation. Accordingly, the model fitness of PSC was also acceptable (0.06, 0.07, and 0.04). Second, although a series of sensitivity analyses have been conducted, we cannot guarantee that each SNP site meets the three basic conditions as instrumental variables. Considering the known confounding factors, we checked the confounders including smoking, drinking, and obesity and eliminated relative IVs. Admittedly, the influence of unknown possible confounders inevitably affects causal inference. Third, the MR model is based on the assumption of a linear effect association between exposure and outcome. Limited by the summary statistics, we did not perform a non-linearity of the association, which may be appropriate in some cases. Lastly, we found statistical heterogeneity between determined IV estimates, which may require further discussion (Supplementary Tables S4–S6).

## Conclusion

MR analyses support the positive causal effect of IBD (including UC and CD) on PSC in the European population. Diagnostics and therapy improvement for PSC as well as the prevention of IBD should be promoted in clinical practice.

## Data Availability Statement

The original contributions presented in the study are included in the article/Supplementary Material, further inquiries can be directed to the corresponding author/s.

## Author Contributions

XW and JC conceptualized and designed the study. YX, XC, and YS collected and analyzed the data in the study. YX and JC drafted the manuscript. All authors contributed to this article and approved the submitted version.

## Conflict of Interest

The authors declare that the research was conducted in the absence of any commercial or financial relationships that could be construed as a potential conflict of interest.
