# Supplementary material for: Causal Linkage Between Inflammatory Bowel Disease and Primary Sclerosing Cholangitis: A Two-Sample Mendelian Randomization Analysis
Source: Front Genet. 2021 Mar 18;12:649376. doi: 10.3389/fgene.2021.649376 (PMC8012893; doi:10.3389/fgene.2021.649376)
Supplement: Supplementary file 1 [file Data_Sheet_1.docx]

Figure S1. MR leave-one-out sensitivity analysis for SNPs of IBD

Figure S2. MR leave-one-out sensitivity analysis for SNPs of UC

Figure S3. MR leave-one-out sensitivity analysis for SNPs of CD

Figure S4. Single SNPs used in the Mendelian randomization analysis of the effects of IBD on PSC.

Figure S5. Single SNPs used in the Mendelian randomization analysis of the effects of UC on PSC.

Figure S6. Single SNPs used in the Mendelian randomization analysis of the effects of CD on PSC.

Figure S7. Funel plot of single SNPs used in the Mendelian randomization analysis of the effects of IBD on PSC.

Figure S8. Funel plot of single SNPs used in the Mendelian randomization analysis of the effects of UC on PSC.

Figure S9. Funel plot of single SNPs used in the Mendelian randomization analysis of the effects of CD on PSC.

Figure S10. MR estimates from each method of assessing the causal effects of IBD on PSC.

Figure S11. MR estimates from each method of assessing the causal effects of UC on PSC.

Figure S12. MR estimates from each method of assessing the causal effects of CD on PSC.


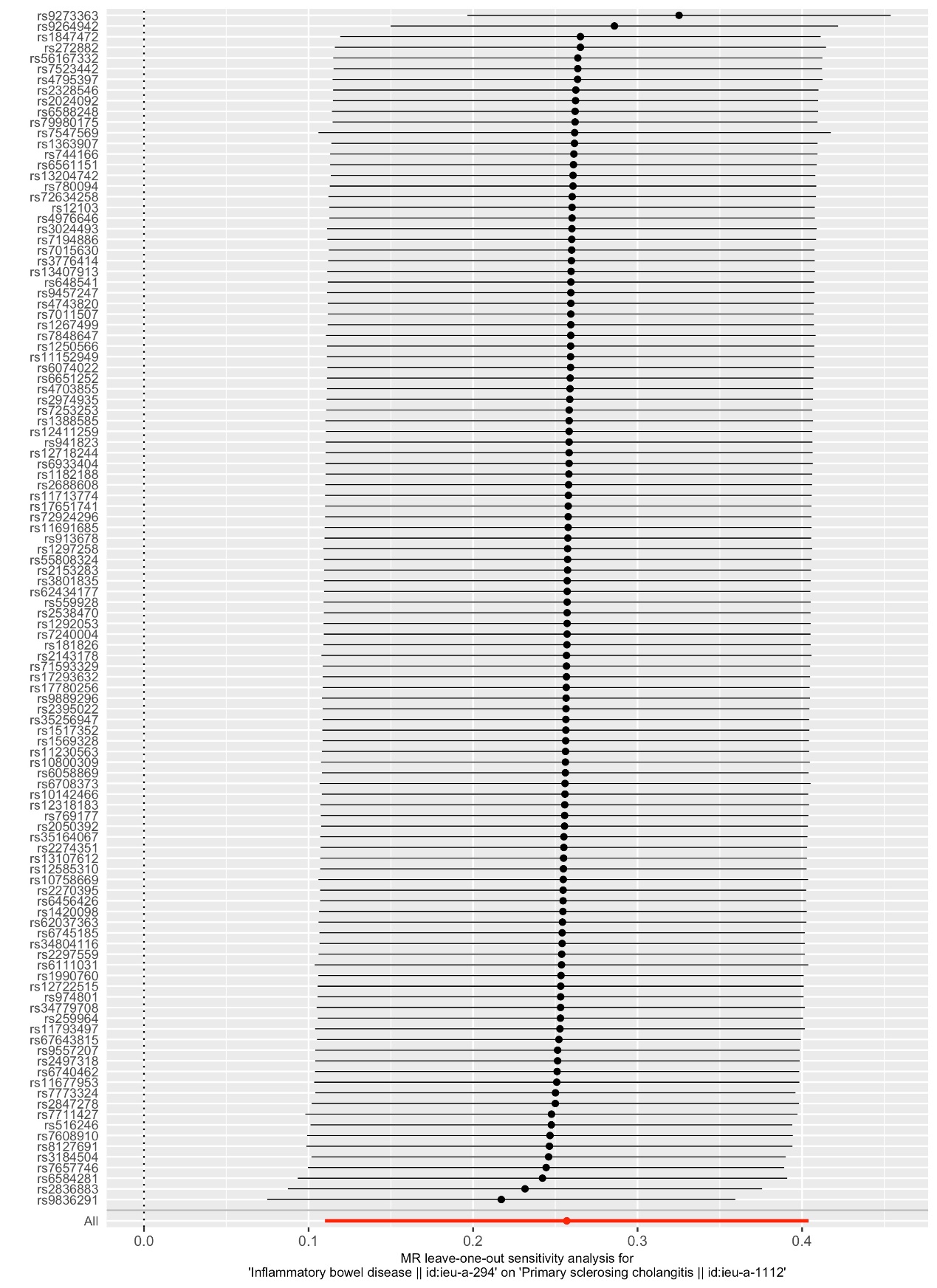


**Figure** S1. MR leave-one-out sensitivity analysis for SNPs of IBD


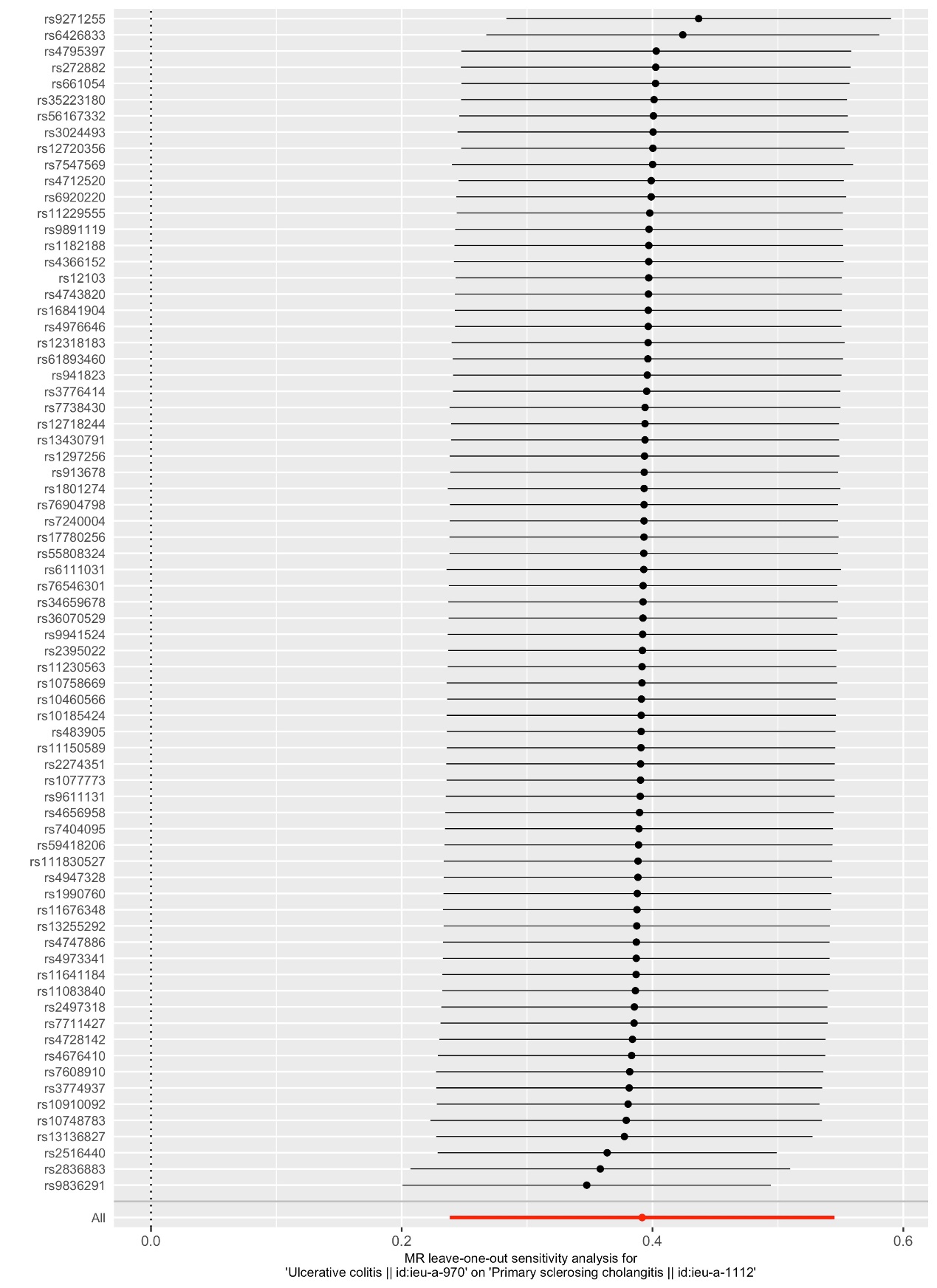


**Figure** S2. MR leave-one-out sensitivity analysis for SNPs of UC


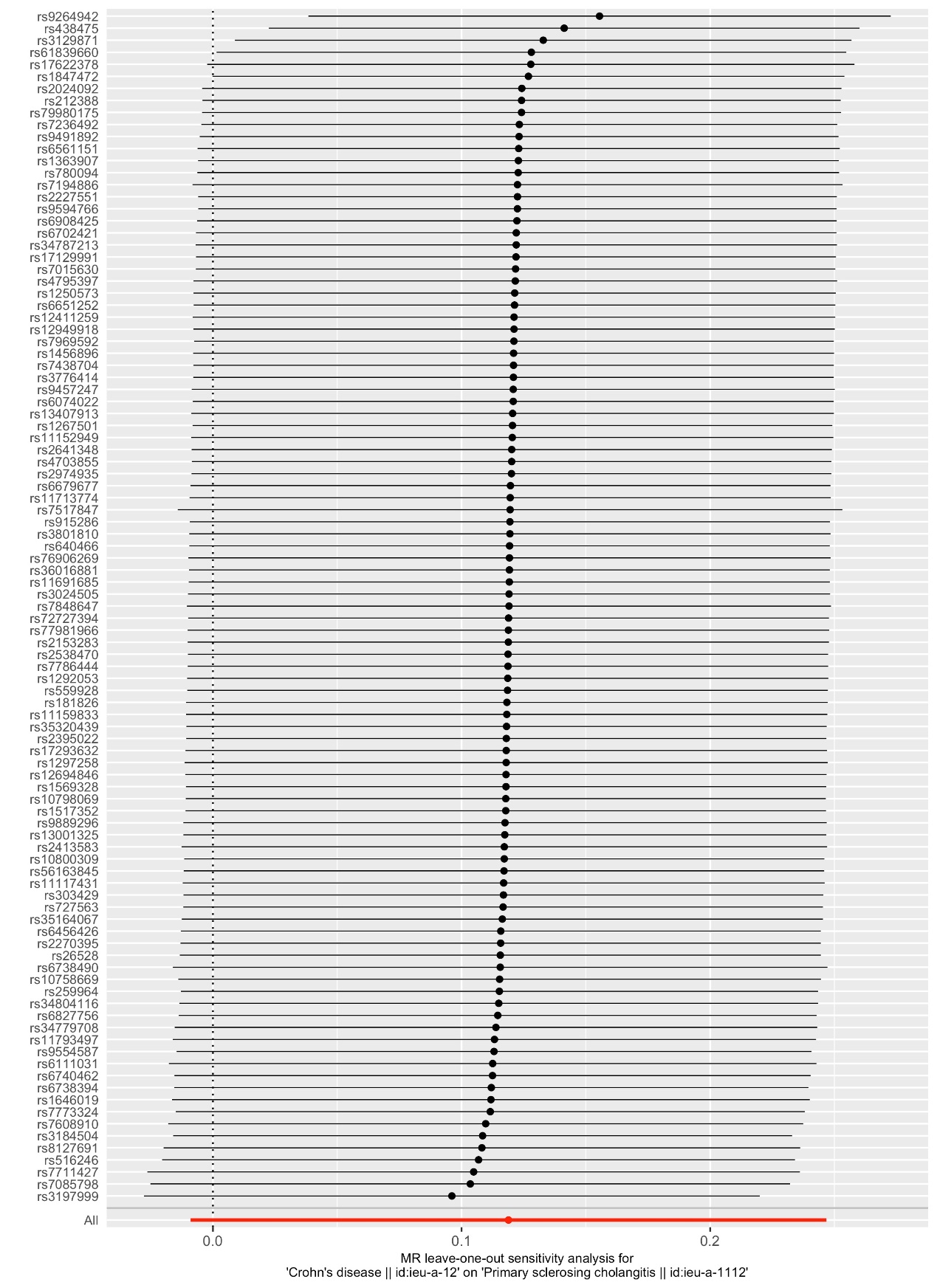


**Figure** S3. MR leave-one-out sensitivity analysis for SNPs of CD


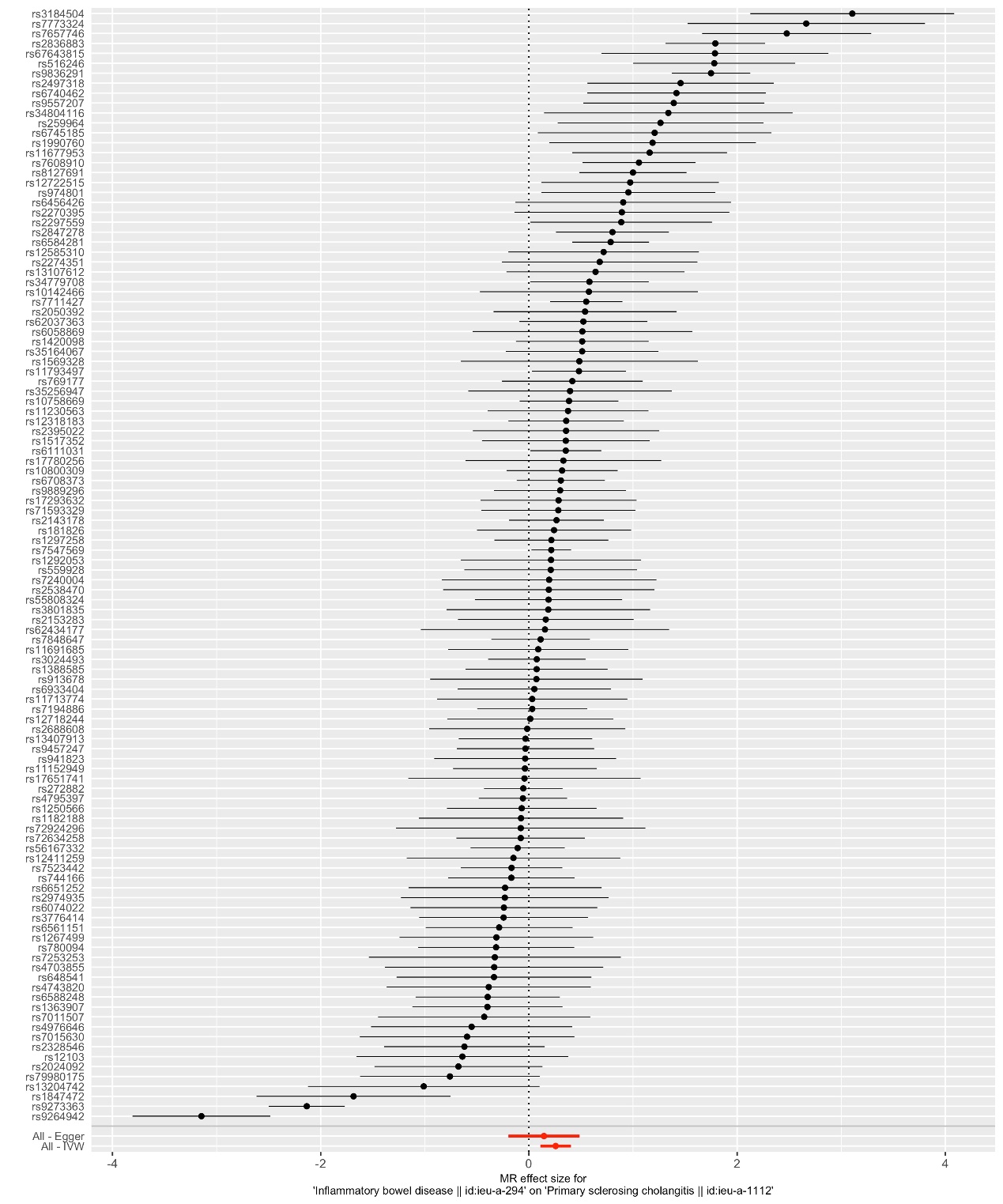


**Figure S4**. Single SNPs used in the Mendelian randomization analysis of the effects of IBD on PSC.
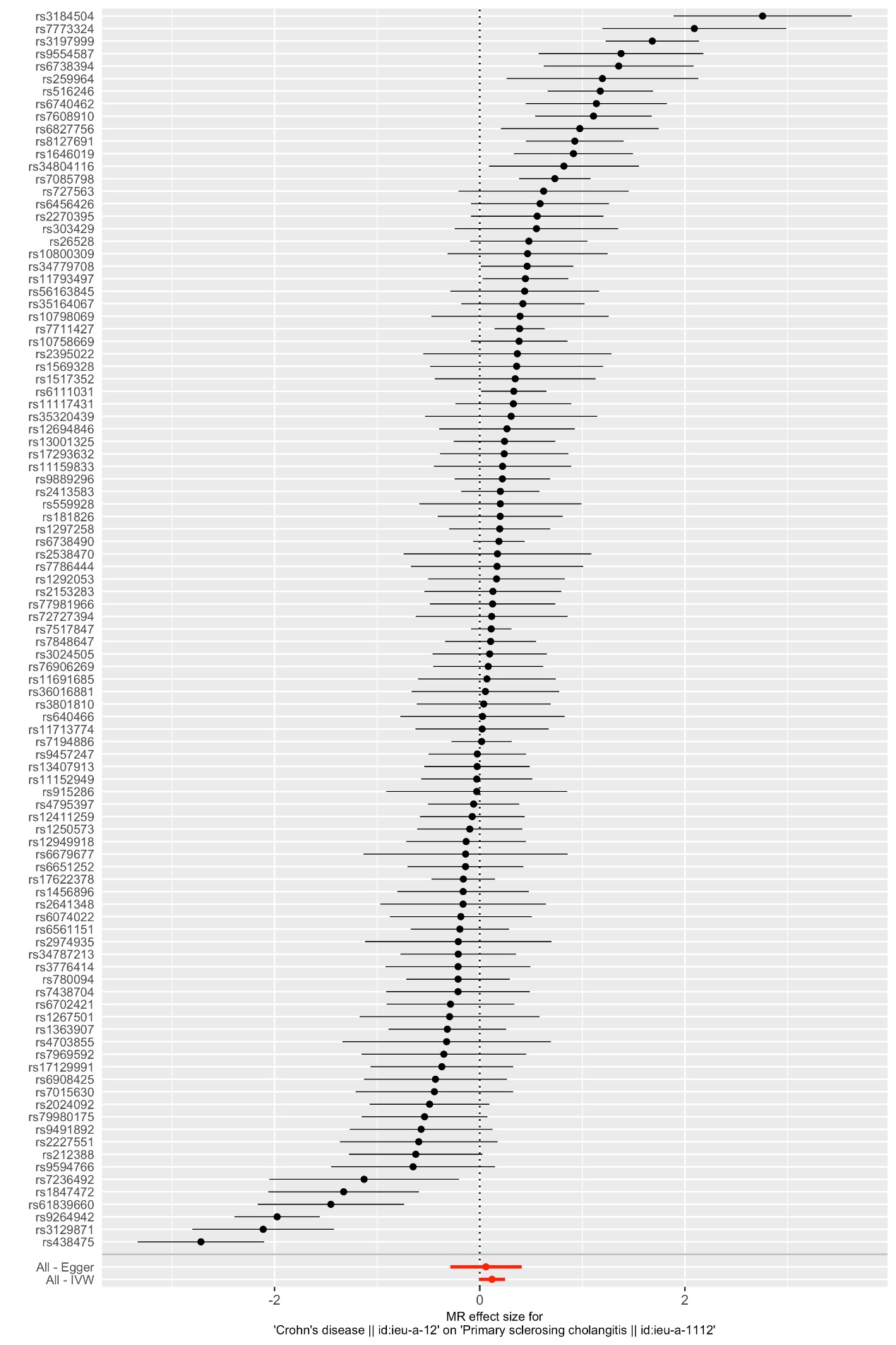


**Figure S5**. Single SNPs used in the Mendelian randomization analysis of the effects of UC on PSC.
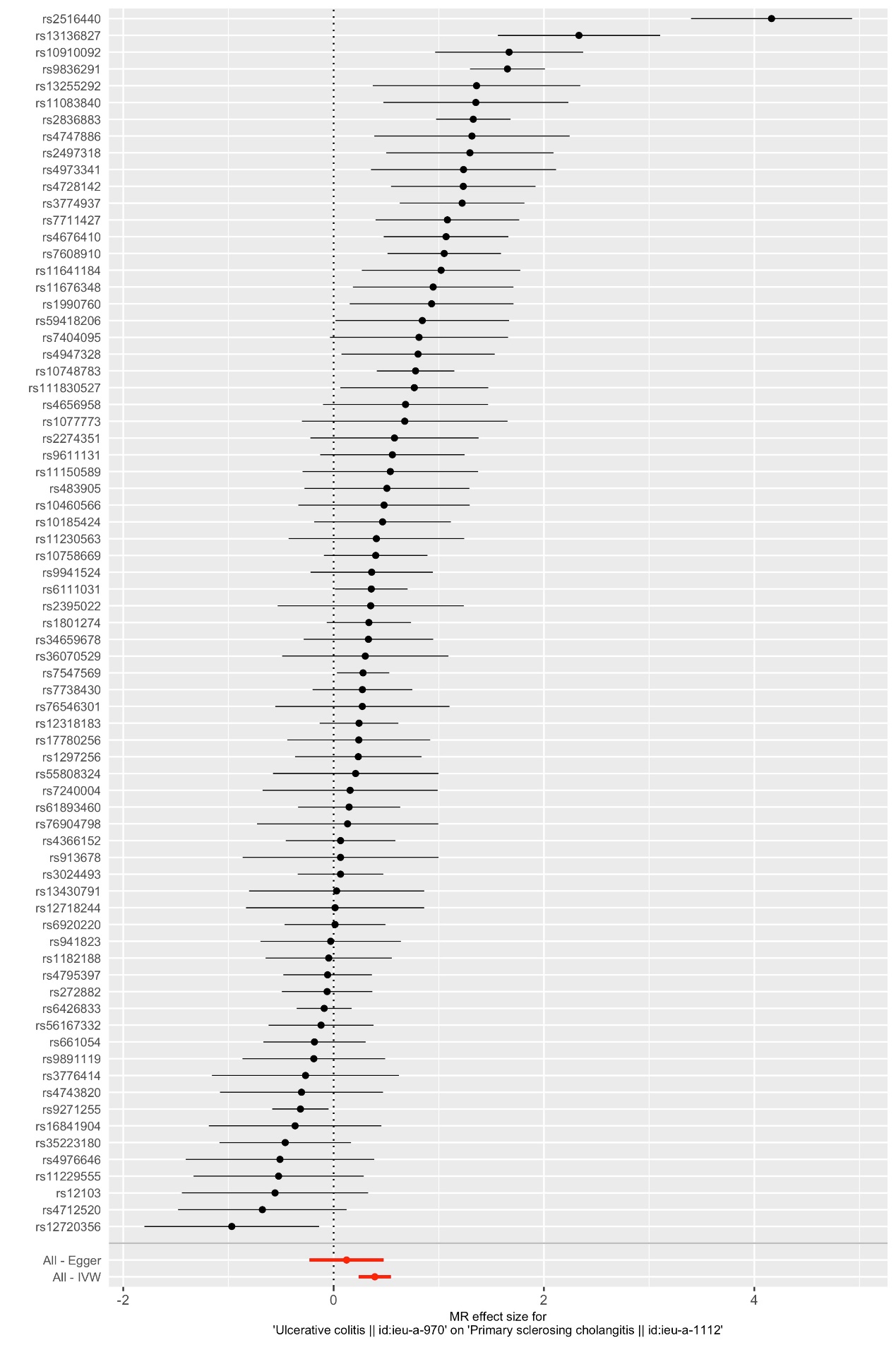


**Figure S6**. Single SNPs used in the Mendelian randomization analysis of the effects of CD on PSC.


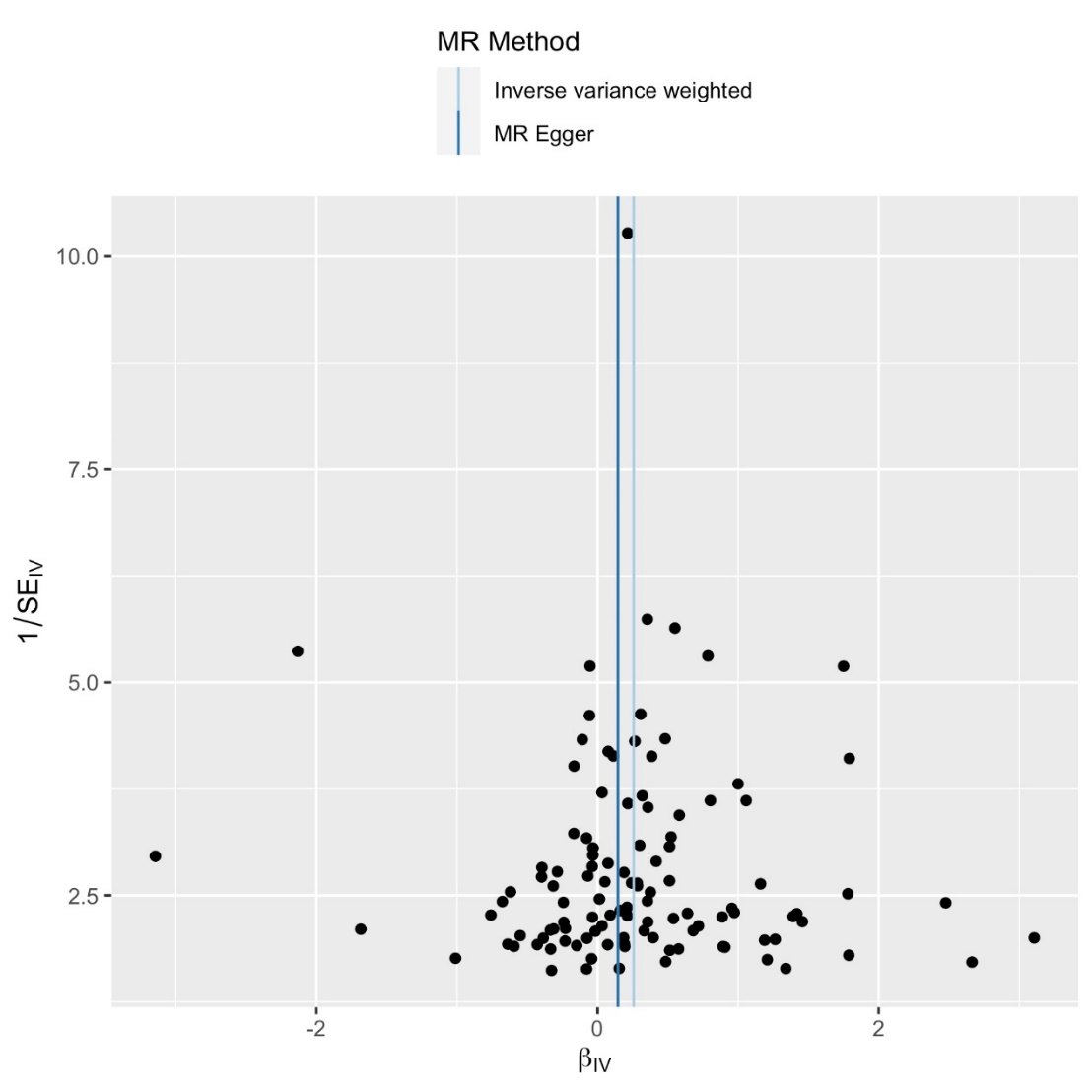


**Figure S7**. Funel plot of single SNPs used in the Mendelian randomization analysis of the effects of IBD on PSC.


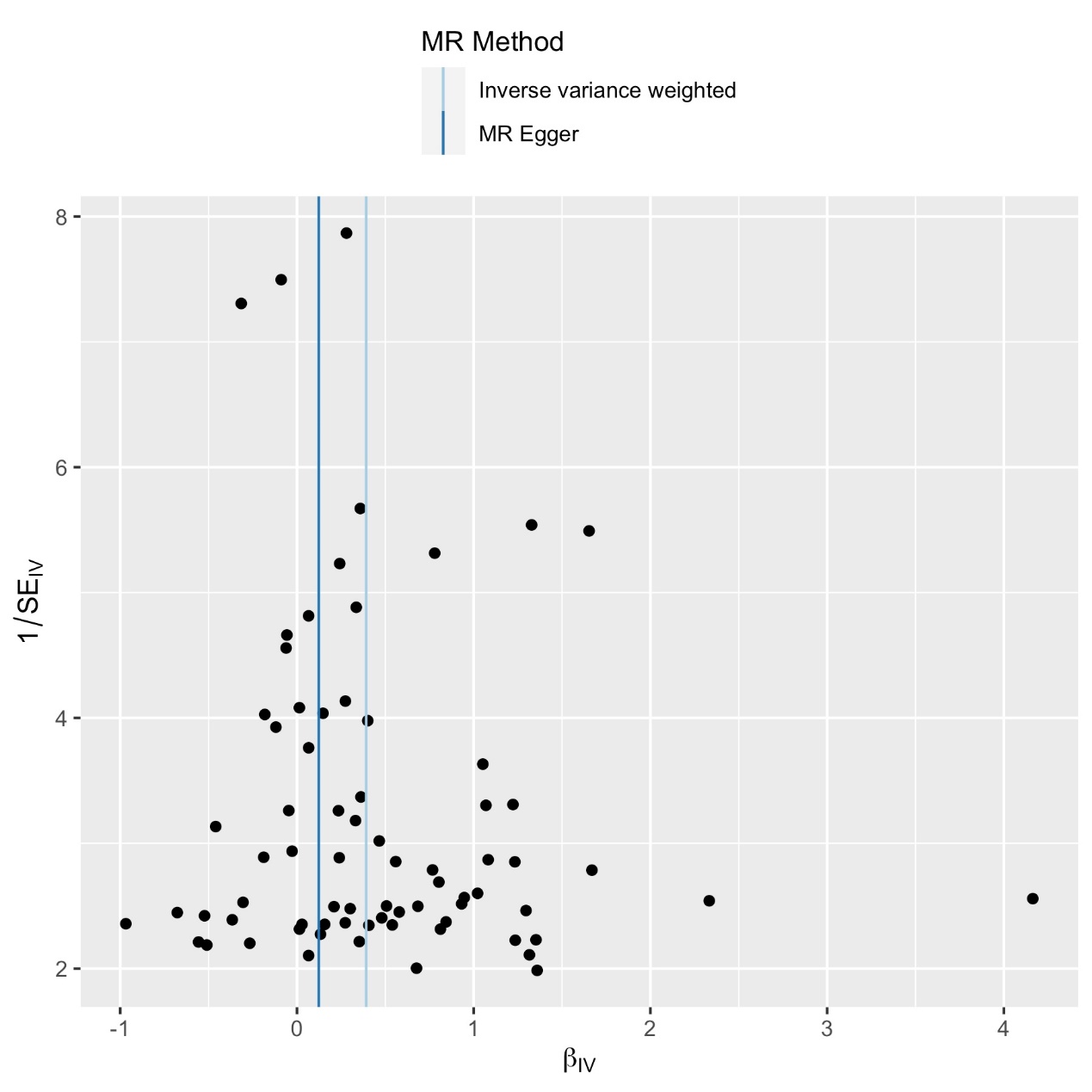


**Figure S8**. Funel plot of single SNPs used in the Mendelian randomization analysis of the effects of UC on PSC.


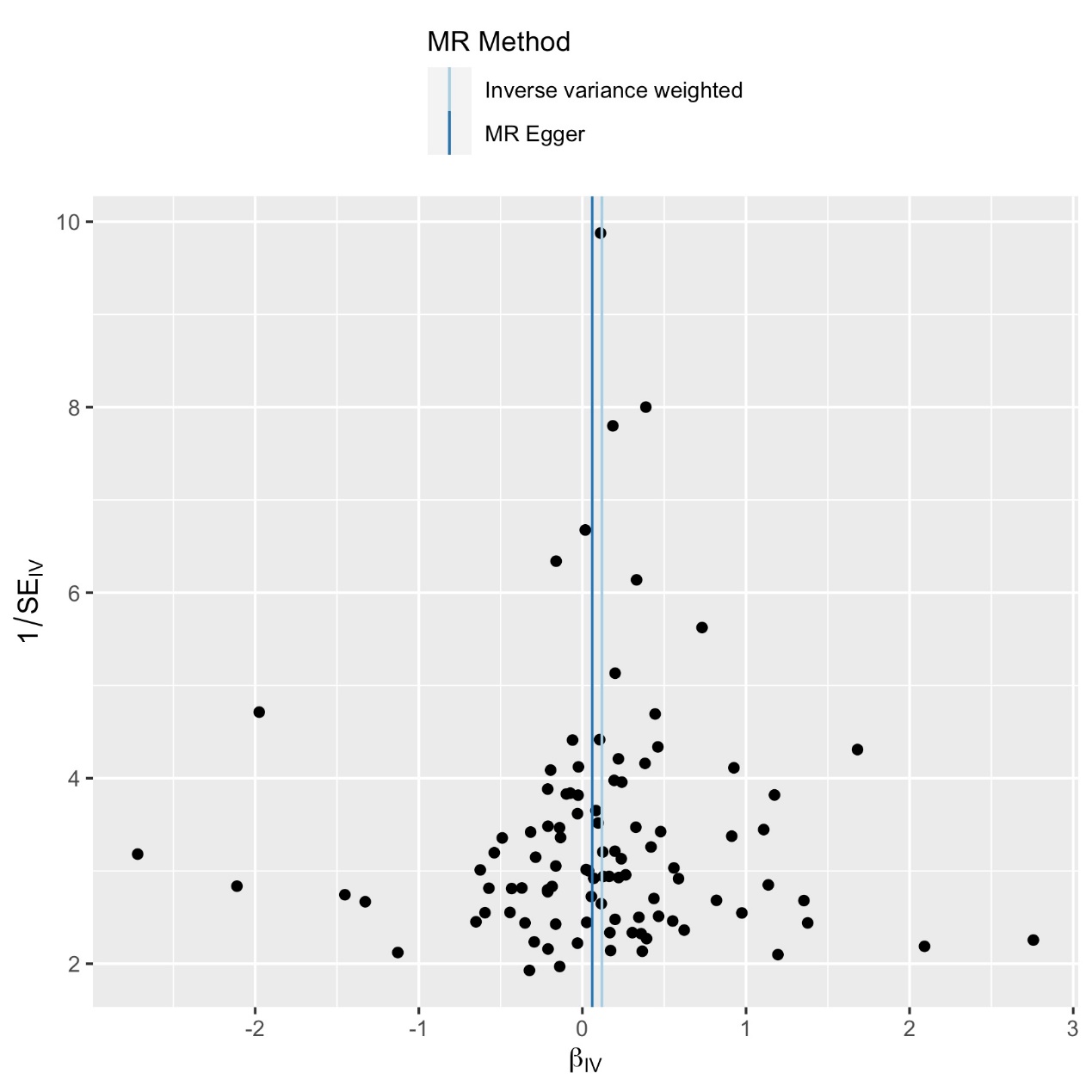


**Figure S9**. Funel plot of single SNPs used in the Mendelian randomization analysis of the effects of CD on PSC.


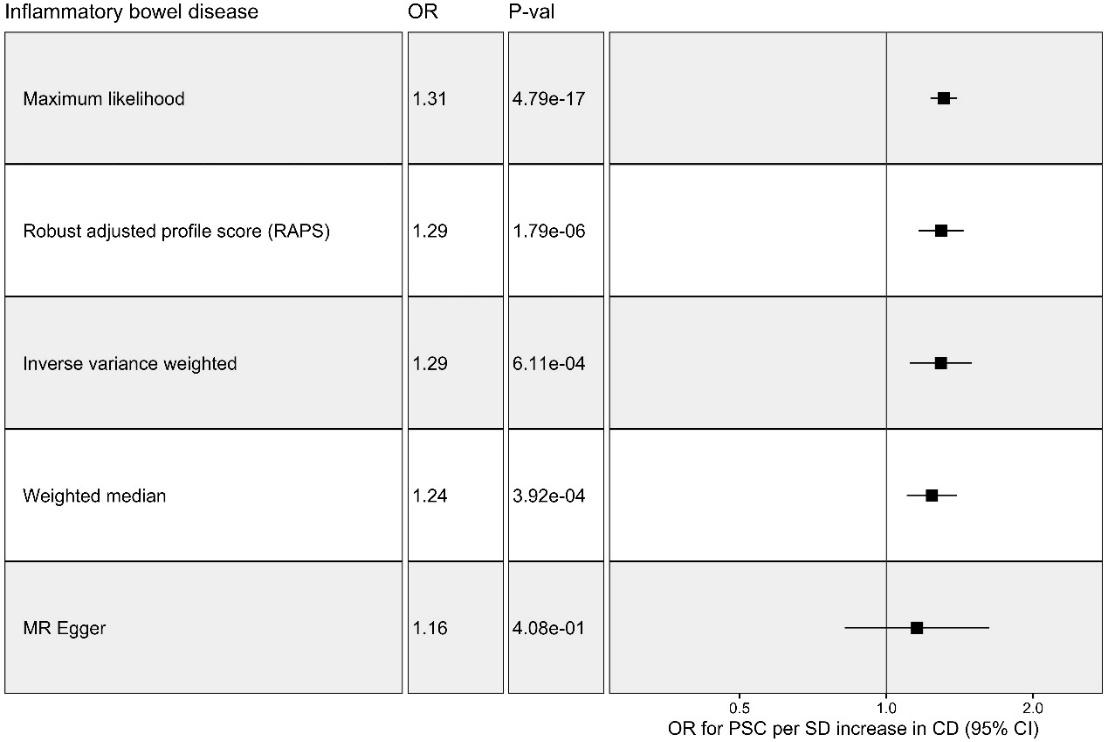


**Figure S10**. MR estimates from each method of assessing the causal effects of IBD on PSC.


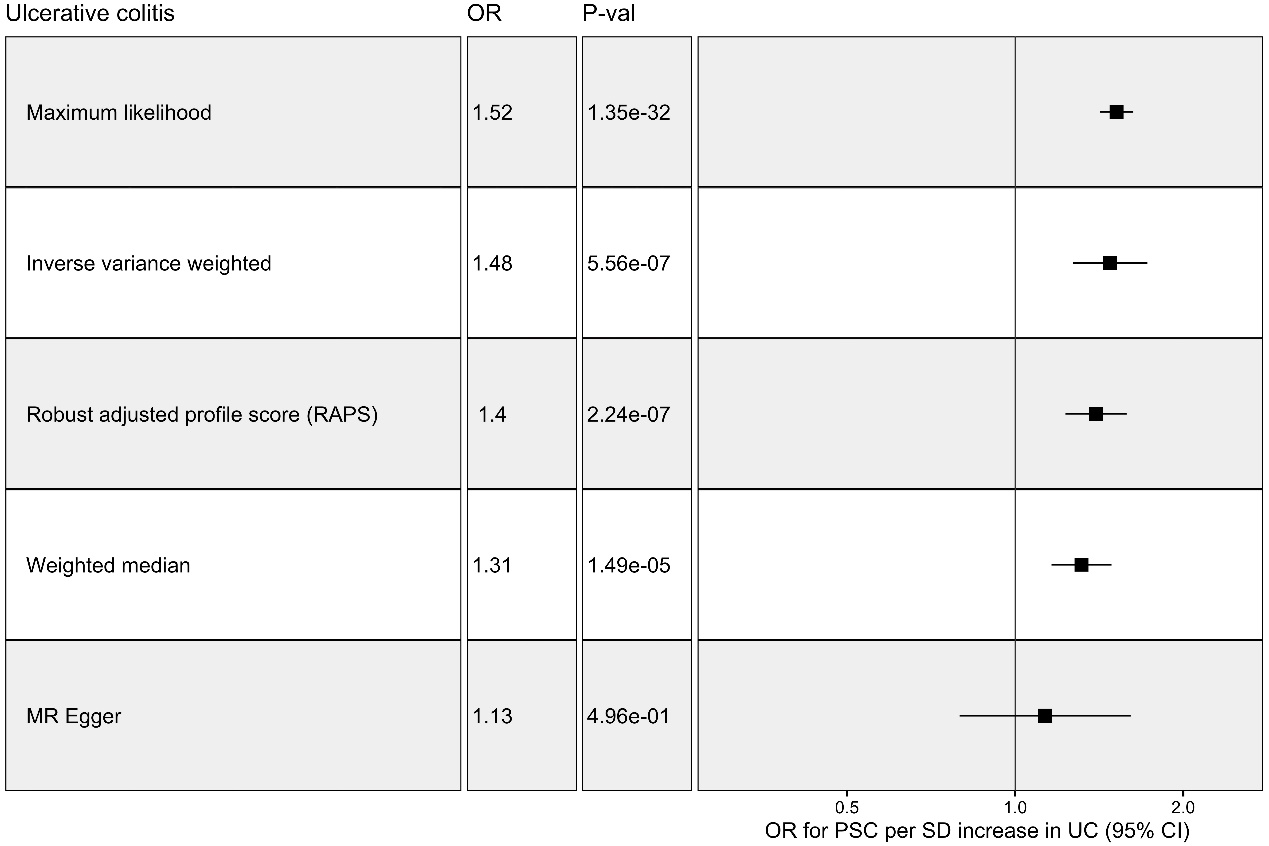


**Figure S11**. MR estimates from each method of assessing the causal effects of UC on PSC.


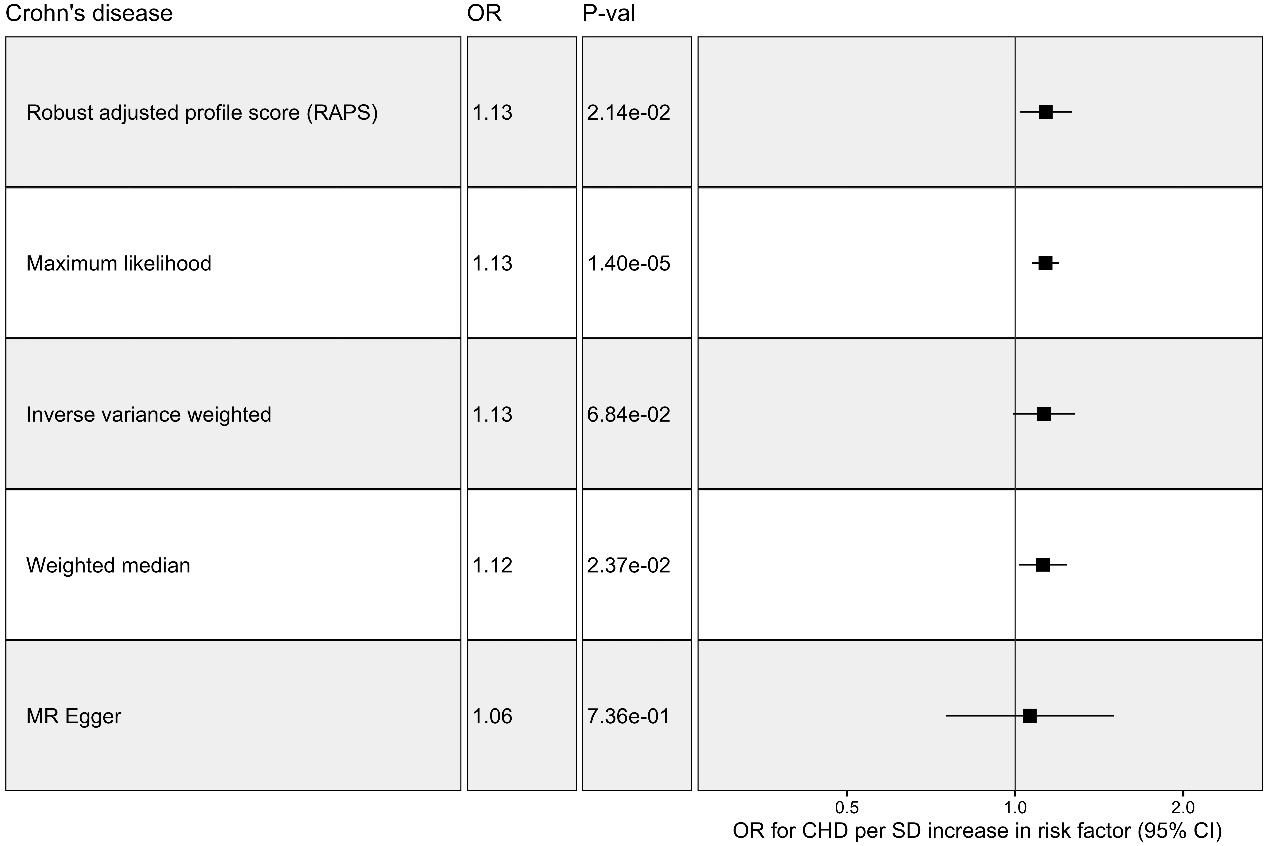


**Figure S12**. MR estimates from each method of assessing the causal effects of CD on PSC.
